# Random Quantum Ising Model with Three-Spin Couplings

**DOI:** 10.3390/e26080709

**Published:** 2024-08-21

**Authors:** Ferenc Iglói, Yu-Cheng Lin

**Affiliations:** 1 Wigner Research Centre for Physics, Institute for Solid State Physics and Optics, H-1525 Budapest, Hungary; 2 Institute of Theoretical Physics, University of Szeged, H-6720 Szeged, Hungary; 3 Graduate Institute of Applied Physics, National Chengchi University, Taipei 11605, Taiwan; yc.lin@nccu.edu.tw

**Keywords:** disordered systems, critical phenomena, renormalization group, infinite disorder fixed point

## Abstract

We apply a real-space block renormalization group approach to study the critical properties of the random transverse-field Ising spin chain with multispin interactions. First, we recover the known properties of the traditional model with two-spin interactions by applying the renormalization approach for the arbitrary size of the block. For the model with three-spin couplings, we calculate the critical point and demonstrate that the phase transition is controlled by an infinite disorder fixed point. We have determined the typical correlation-length critical exponent, which seems to be different from that of the random transverse Ising chain with nearest-neighbor couplings. Thus, this model represents a new infinite disorder universality class.

## 1. Introduction

Quantum phase transitions take place at zero temperature by varying a control parameter, such as the strength of a transverse field, and are signaled by a singularity in the ground state of the system [1]. Quantum phase transitions occur also in one space dimension, and the corresponding singularity is often related to the temperature-driven critical singularities in two-dimensional classical systems [2]. Disorder is an inevitable feature of real materials, and it could have a very profound effect on the critical behavior of the systems [3], and the effect of the disorder is particularly strong at a quantum critical point. The theoretical description of a random quantum phase transition is very challenging since the combined effect of disorder and quantum fluctuations, together with strong correlations, have to be considered at the same time. For some models, this type of investigation can be performed by a so called strong disorder renormalization group (SDRG) method [4,5]. In the SDRG calculation, local degrees of freedom with a large excitation energy are successively eliminated and new parameters are calculated perturbatively for the remaining degrees of freedom. In a class of models, the distribution of the renormalized parameters broaden without limit, and the random phase transition is controlled by a so-called infinite disorder fixed point (IDFP) [6]. At an IDFP, disorder fluctuations are overwhelmingly dominant over quantum fluctuations, and the obtained critical properties are expected to be asymptotically exact for large systems.

The SDRG approach was introduced by Ma, Dasgupta and Hu [7,8] and later applied by D. Fisher [9,10] to study the critical properties of the random transverse-field Ising chain. Several presumably exact results have been obtained so that the random model looks to be understood at least at the same level as its nonrandom counterpart. In one dimension, the method has been generalized for other models, resulting in exact solutions [11,12,13,14,15]. In higher dimensions, the method is applied numerically [16,17,18,19,20,21], and different technical simplifications are introduced in order to treat large finite systems [22,23,24]. For the transverse Ising model, it is demonstrated that the critical behavior is controlled by IDFPs even at higher dimensions, and it is expected that the upper critical dimension of the problem is infinite [23,24]. Regarding models with nearest neighbor interaction and having a discrete order parameter, the critical behavior is expected to be the same as that of the transverse Ising model. This result seems to hold also for random stochastic models, such as the random contact process [25,26,27]. For random models with long-range interactions, however, a new type of fixed point is found to control the critical behavior, which is of a conventional disorder type [28,29].

In this paper, we consider another type of transverse Ising model, which has multiple-site product interactions. The Hamiltonian is defined as

(1) $$H^{\left(m\right)}=-\sum_{i}J_{i}\prod_{l=0}^{m-1}\sigma_{i+l}^{z}-\sum_{i}h_{i}\sigma_{i}^{x}\;,$$

in terms of the Pauli-matrices $\sigma_{i}^{x,z}$ at site *i*. Here, the interactions $J_{i}$ and the transverse fields $h_{i}$ are both independent random variables. This model in a pure (nonrandom) case was introduced by Turban [30] and independently by Penson et al. [31]. The special case m=2 is the standard transverse Ising chain with nearest neighbor interaction. According to numerical studies, the pure m=3 model has a second-order quantum phase transition, which belongs to the universality class of the 4-state Potts model [32,33,34,35,36,37]. At the critical point, there are logarithmic corrections that are probably in the same form as in the 4-state Potts model [38,39]. For m≥4, the phase-transition in the pure model is of first order.

In this paper, we are going to study the phase transition in the disordered model with random $J_{i}>0$ and $h_{i}>0$. We do not specify the form of the disorder distributions. We assume that these are not singular so that the first and second moments of the log-variables are finite. In this respect, we have detailed information about the m=2 model [4,5], but the models with m>2 have not been investigated yet. Here, we study the model with m=3 and consider random positive three-spin couplings and random positive transverse fields. Due to the different types of local interactions, the application of the standard SDRG method [4,5] exhibits problems: by eliminating a strong three-spin coupling, several new renormalized interactions will be generated, depending on the neighborhood of the eliminated coupling; in this case, one can not stop the proliferation of the renormalized parameters during the renormalization procedure. Therefore, we chose a different approach and used a block renormalization group method, which was introduced to the pure m=2 Ising chain by Fernandez-Pacheco [40]. This type of renormalization preserves the self-duality of the model and reproduces the exact critical point and the value of the exact correlation length exponent, ν(m=2)=1. Later, the method was used for other quantum spin chains [41,42,43,44,45,46,47] and was generalized for higher dimensions [48,49]. For the random transverse Ising model with nearest neighbor couplings (m=2), the method was applied by Miyazaki and Nishimore [50], as well as by Cécile Monthus [51], both in one and higher dimensions.

Our paper is organized as follows. In Section 2, we introduce the basic idea of the block renormalization approach and present the duality transformation of the model. In Section 3, the method is applied to the m=2 model by using the arbitrary large size of the block, while in Section 4, it is applied to the m=3 model with a block size b=2. In Section 5, we close our paper with a discussion.

## 2. Basic Idea of Block Renormalization

In the block renormalization method [52,53] the spins at sites i=nb+1 with n=0,1,2,… and b=2,3,… are fixed at arbitrary positions, while the intermediate spins are integrated out. Here, *b* sets the scale factor, which is the size of the blocks. In this way, the Hamiltonian is divided into two parts:

(2) $$H^{\left(m\right)}=H_{0}^{\left(m\right)}+V^{\left(m\right)}\;,$$

where $H_{0}^{\left(m\right)}$ represents blocks with the intra-block terms, and $V^{\left(m\right)}$ is the perturbation that contains the transverse field acting on the selected spins and the couplings that couple the neighboring blocks. The block-Hamiltonians are solved either analytically or numerically and the lowest levels are retained and identified as the states of the block-spin variable. At the same time, the renormalized values of the inter-block terms are obtained in a (first-order) perturbative way.

In the use of the block renormalization approach, it is useful if the models have duality properties; in which case, the number of parameters generally does not increase during renormalization. The Hamiltonian in Equation (4) indeed has such a symmetry. Following the method by Turban [30], one can define a set of variables:

(3) $$\begin{array}{cc} \mu_{i}^{x} & =\prod_{l=0}^{m-1}\sigma_{i+l}^{z}\\ \mu_{i}^{z} & =\prod_{n=0}^{\infty }\sigma_{i-nm}^{x}\sigma_{i-nm-1}^{x}\;, \end{array}$$

that satisfy the Pauli spin algebra. In terms of these new variables, the Hamiltonian is expressed as

(4) $$H^{\left(m\right)}=-\sum_{i}h_{i}\prod_{l=0}^{m-1}\mu_{i-l}^{z}-\sum_{i}J_{i}\mu_{i}^{x}\;,$$

which is in the same form as that in Equation (1) with the correspondences $J_{i}\to h_{i+m-1}$ and $h_{i}\to J_{i}$. It follows that the pure model with $J_{i}=J,h_{i}=h,\forall i$ is self-dual and the self-dual point J/h=1 corresponds to the phase-transition point.

In the following, we first present the block renormalization for the m=2 model for arbitrary size of the block [47], then we consider the model with m=3 and solve the renormalization approach for a block of two sites.

## 3. Block Renormalization Approach of the m=2 Model

Here, we first turn the spin variables $\sigma_{i}^{x}↔\sigma_{i}^{z}$, and for convenience, the sites are relabeled as i=(j,α), where j=1,2,… labels the blocks and α=0,1,…,b−1 labels sites in a block. For the m=2 model, the intra-block Hamiltonian is

(5) $$H_{0}^{\left(2\right)}=-\sum_{j}\sum_{\alpha =0}^{b-2}J_{j,\alpha +1}\sigma_{j,\alpha }^{x}\sigma_{j,\alpha +1}^{x}-\sum_{j}\sum_{\alpha =1}^{b-1}h_{j,\alpha }\sigma_{j,\alpha }^{z}\;,$$

while the perturbation is given by

(6) $$V^{\left(2\right)}=-\sum_{j}J_{j-1,b-1}\sigma_{j-1,b-1}^{x}\sigma_{j,0}^{x}-\sum_{j}h_{j,0}\sigma_{j,0}^{z}\;.$$

The leftmost spin of a block is fixed, thus the *x*-component of this spin is ±1, which fixes the sign of the magnetization at the other sites of the block, too. We use this sign to characterize the state of the block. Solving the ground state of the block both with + and − leftmost spins, the interaction energy between two neighboring blocks is given in first-order perturbation theory as

(7) $$\epsilon_{j}=-J_{j,b-1}\langle \sigma_{j,b-1}^{x}\rangle \langle \sigma_{j+1,0}^{x}\rangle =-J_{j}^{R}\Sigma_{j}^{x}\Sigma_{j+1}^{x}\;,$$

where $\langle \sigma_{j+1,0}^{x}\rangle =\pm 1$ is fixed, and $\langle \sigma_{j,b-1}^{x}\rangle =m_{j}^{\left(b\right)}$ is the expectation value of the end-spin magnetization in the *j*-th block of length *b*. The block-spin variables are $\Sigma_{j}^{x}=\pm 1$ and $\Sigma_{j+1}^{x}=\pm 1$, thus the renormalized value of the coupling is

(8) $$J_{j}^{R}=J_{j,b-1}m_{j}^{\left(b\right)}\;.$$

The end-spin magnetization can be calculated exactly [47,54,55]:

(9) $$m_{j}^{\left(b\right)}={\left[1+\sum_{\alpha =1}^{b-1}\prod_{k=1}^{\alpha }{\left(\frac{h_{j,b-k}}{J_{j,b-k-1}}\right)}^{2}\right]}^{-1/2}\;,$$

which, in the simplest case with b=2, is given by

(10) $$m_{j}^{\left(2\right)}={\left[1+{\left(\frac{h_{j,1}}{J_{j,0}}\right)}^{2}\right]}^{-1/2}\;.$$

The renormalized value of the transverse field is obtained through duality, which amounts to interchange couplings and fields, and also the two ends of the block leading to

(11) $$h_{j}^{R}=h_{j,0}{\tilde{m}}_{j}^{\left(b\right)}\;,$$

with

(12) $${\tilde{m}}_{j}^{\left(b\right)}={\left[1+\sum_{\alpha =1}^{b-1}\prod_{k=1}^{\alpha }{\left(\frac{J_{j,k-1}}{h_{j,k}}\right)}^{2}\right]}^{-1/2}\;,$$

and

(13) $${\tilde{m}}_{j}^{\left(2\right)}={\left[1+{\left(\frac{J_{j,0}}{h_{j,1}}\right)}^{2}\right]}^{-1/2}\;.$$

Let us consider the ratio

(14) $$K_{j,\alpha }=\frac{J_{j,\alpha }}{h_{j,\alpha }}\;,$$

and calculate its value with the renormalized parameters, which are given by

(15) $$K_{j}^{R}=\frac{J_{j}^{R}}{h_{j}^{R}}=\frac{\prod_{\alpha =0}^{b-1}J_{j,\alpha }}{\prod_{\alpha =0}^{b-1}h_{j,\alpha }}=\prod_{\alpha =0}^{b-1}K_{j,\alpha }\;.$$

### 3.1. Pure Model

For the pure model with $K=K_{j,\alpha },\;\forall j,\alpha$, Equation (15) leads to the simple relation [47]:

(16) $$K^{R}=K^{b}$$

having the fixed point $K^{*}={\left(J/h\right)}^{*}=1$, which corresponds to the self-dual point of the system. Furthermore, the thermal eigenvalue of the transformation is $\lambda_{t}=b$ and the correlation-length critical exponent is $\nu^{\mathrm{pure}}=\ln b$/$\ln\lambda_{t}=1$. This is the exact value, and in this way, it has been obtained for any value of *b*.

### 3.2. Random Model

Now we turn to the disordered model with random couplings and random fields. The renormalization equation in Equation (15) has a simple addition form in terms of the log-variables:

(17) $$\ln K_{j}^{R}=\sum_{\alpha =0}^{b-1}\ln K_{j,\alpha }\;.$$

Interestingly, there are two scale factors, $\tilde{b}<b$, such that ${\tilde{b}}^{N}=b$. Repeating the iterations with the scale factor $\tilde{b}$ *N*-times (N=2,3,…), the form of the renormalized *K*-parameter is the same as if the renormalization is performed with a scale factor *b* in one step.

According to the central-limit theorem, Equation (17) in the large-*b* limit has the asymptotic form

(18) $$\begin{array}{cc} \ln K_{b}^{R} & =b\left[\bar{\ln J_{\alpha }-\ln h_{\alpha }}\right]\\ \; & +b^{1/2}{\left[Var\left(\ln J_{\alpha }\right)+Var\left(\ln h_{\alpha }\right)\right]}^{1/2}v \end{array}$$

where $\bar{x}$ and Var(x) denote the mean value and the variance of the random variable *x*, respectively, and *v* is a Gaussian random variable with mean zero and variance unity. Introducing the quantum control parameter

(19) $$\delta =\frac{\bar{\ln J_{\alpha }}-\bar{\ln h_{\alpha }}}{Var\left(\ln J_{\alpha }\right)+Var\left(\ln h_{\alpha }\right)}\;,$$

we have

(20) $$\begin{array}{cc} \ln K_{b}^{R} & =b\delta +b^{1/2}{\left[Var\left(\ln J_{\alpha }\right)+Var\left(\ln h_{\alpha }\right)\right]}^{-1/2}v\;. \end{array}$$

The first term in the above equation characterizes the divergence of the typical correlation length, since by definition $b∼\xi_{\mathrm{typ}}∼\delta^{-\nu_{typ}}$ near criticality. From Equation (20), we obtain the typical correlation length exponent:

(21) $$\nu_{\mathrm{typ}}=1\;.$$

At the critical point (δ=0), the fluctuations in the log-couplings grow as $b^{1/2}$, which defines the log-excitation energy:lnϵ. The corresponding scaling form is $\ln\epsilon ∼b^{\psi }$ with the critical exponent:

(22) $$\psi =\frac{1}{2}\;.$$

This type of dynamic is relevant at an infinite disorder fixed point.

The combination of the first and the second terms in Equation (20) with $b\delta \approx -b^{1/2}$${\left[Var\left(\ln J_{\alpha }\right)+Var\left(\ln h_{\alpha }\right)\right]}^{-1/2}$ will result in the vanishing of the leading contribution of these terms, which is connected to a finite-size correlation exponent [51], $\nu_{\mathrm{FS}}$ defined by $\delta ∼b^{-1/\nu_{\mathrm{FS}}}$ and given by

(23) $$\nu_{\mathrm{FS}}=2\;.$$

These results follow directly from the RG equations in Equations (8) or (11) and from the known scaling properties of the end-spin magnetization [55]. We can also recover known results in the off-critical region, in the so-called Griffith’s phase [56,57]. Using Equations (11) and (12) we express the inverse square of the excitation energy as

(24) $$\frac{1}{\epsilon^{2}}\equiv S=1+\sum_{\alpha =1}^{b-1}\prod_{k=1}^{\alpha }{\left(\frac{J_{j,k-1}}{h_{j,k}}\right)}^{2}\;,$$

which in the limit b→∞ is a Kesten variable [58], which has a tail distribution:

(25) $$P\left(S\right)∼\frac{1}{S^{1+\Delta }}\;,$$

for S≫1 with the exponent which is the positive root of the equation [59,60,61]:

(26) $$\bar{{\left(\frac{J^{2}}{h^{2}}\right)}^{\Delta }}=1\;.$$

Here, Δ is related to the dynamical exponent *z* in the Griffith’s phase via

(27) $$z=\frac{1}{2\Delta }\;,$$

so that length and excitation energy are related as

(28) $$\epsilon ∼b^{-z}\;,$$

and the asymptotic distribution of the log-excitation energy is given by

(29) $$P\left(\ln\epsilon \right)∼\epsilon^{1/z}\;$$

for ϵ≪1.

## 4. Block Renormalization Approach of the m=3 Model

For the m=3 model, the Hamiltonian is split as

(30) $$H_{0}^{\left(3\right)}=-\sum_{j}\sum_{\alpha =0}^{b-2}J_{j,\alpha +1}\sigma_{j,\alpha }^{x}\sigma_{j,\alpha +1}^{x}\sigma_{j,\alpha +2}^{x}-\sum_{j}\sum_{\alpha =1}^{b-1}h_{j,\alpha }\sigma_{j,\alpha }^{z}\;,$$

and

(31) $$V^{\left(3\right)}=-\sum_{j}J_{j,0}\sigma_{j-1,b-1}^{x}\sigma_{j,0}^{x}\sigma_{j,1}^{x}-\sum_{j}h_{j,0}\sigma_{j,0}^{z}\;.$$

**Figure 1:**
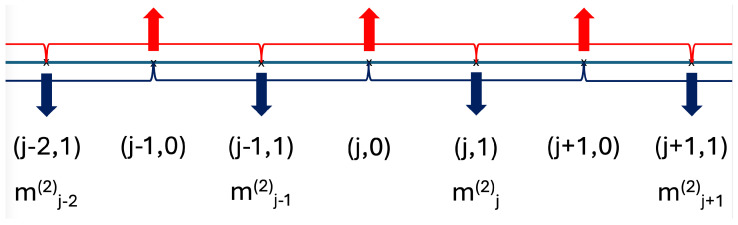
Illustration of the division of the Hamiltonian into the unperturbed part ($H_{0}$: ⇓, $\underset{︸}{\;}$) and the perturbation (V: ⇑, $\overset{︷}{\;}$), for transverse fields and three-spin couplings, respectively, for m=3 and b=2. The magnetization in the *j*-th block is denoted by $m_{j}^{\left(2\right)}$, see in Equation (33).

Here, we restrict ourselves to the case where the scale factor b=2, which allows the RG method to be solved analytically. For larger blocks b>2, the calculations are more complicated due to the presence of three-spin interactions. The way the Hamiltonian with m=3 is divided into two parts for b=2 is illustrated in Figure 1.

Solving the ground state of the *j*th-block, the interaction energy between neighboring blocks in first-order perturbation theory is given by

(32) $$\epsilon_{j}=-J_{j,0}\langle \sigma_{j-1,1}^{x}\rangle \langle \sigma_{j,0}^{x}\rangle \langle \sigma_{j,1}^{x}\rangle =-J_{j}^{R}\Sigma_{j}^{x}\sigma_{j,0}^{x}\Sigma_{j+1}^{x}\;,$$

where $\langle \sigma_{j,0}^{x}\rangle =\pm 1$ is fixed, $\Sigma_{j}^{x}$ denotes the block spin for the *j*th-block, and $J_{j}^{R}$ the renormalized three-spin coupling. The expectation value $\langle \sigma_{j,1}^{x}\rangle =m_{j}^{\left(2\right)}$ is given by

(33) $$m_{j}^{\left(2\right)}={\left[1+{\left(\frac{h_{j,1}}{J_{j,1}}\right)}^{2}\right]}^{-1/2}\;,$$

and similarly for $\langle \sigma_{j-1,1}^{x}\rangle =m_{j-1}^{\left(2\right)}$. Thus, the renormalized value of the three-spin coupling is given by

(34) $$J_{j}^{R}=J_{j,0}m_{j-1}^{\left(2\right)}m_{j}^{\left(2\right)}\;.$$

The renormalized value of the transverse field is obtained through duality, which amounts to interchange couplings and fields, leading to

(35) $$h_{j}^{R}=h_{j,0}{\tilde{m}}_{j-1}^{\left(2\right)}{\tilde{m}}_{j}^{\left(2\right)}\;,$$

with

(36) $${\tilde{m}}_{j}^{\left(2\right)}={\left[1+{\left(\frac{J_{j,1}}{h_{j,1}}\right)}^{2}\right]}^{-1/2}\;.$$

Considering the ratio defined in Equation (15), we obtain

(37) $$K_{j}^{R}=\frac{J_{j}^{R}}{h_{j}^{R}}=\frac{J_{j-1,1}J_{j,0}J_{j,1}}{h_{j-1,1}h_{j,0}h_{j,1}}=K_{j-1,1}K_{j,0}K_{j,1}\;,$$

as a product of three original ratios.

### 4.1. Pure Model

For the pure model, there is a simple relation [32]:

(38) $$K^{R}=K^{3}\;,$$

having the fixed-point $K^{*}={\left(J/h\right)}^{*}=1$, which is the self-dual point of the system. The thermal eigenvalue is $\lambda_{t}=3$, and the correlation length exponent is $\nu^{pure}=\ln2/$ln3=0.631. This value is not exact but quite close to the expected value of the 4-state Potts model [3]: $\nu^{Potts}=2/3$.

### 4.2. Random Model

For the random model, repeating the renormalization in the next step, we obtain

(39) $$\begin{array}{cc} \; & K_{j}^{R(2)}=K_{j-1}^{R}K_{j}^{R}K_{j+1}^{R}\\ \; & =K_{j-2,1}K_{j-1,0}K_{j-1,1}^{2}K_{j,0}K_{j,1}^{2}K_{j+1,0}K_{j+1,1}\;, \end{array}$$

which contains the product of nine terms, but two terms are represented twice. Let us introduce log-ratios and iterate the renormalization process *n*-times, then we obtain an additive form:

(40) $$\ln K^{R(n)}=\sum_{i=1}^{L(n)}c_{i}^{\left(n\right)}\ln K_{i}\;,$$

where we have used the original notation, *i*, for the sites, and $c_{i}^{\left(n\right)}$ is the multiplicity of the term $K_{i}$. For the first few iterations, the multiplicities are given by

(41) $$\begin{array}{cc} n=1\; & 111\\ n=2\; & 1121211\\ n=3\; & 112132313231211\\ n=4\; & 1121323143525341435253413231211\;, \end{array}$$

which can be generated using the following rules:

(42) $$c_{2^{n}}^{\left(n\right)}=1,\;c_{2^{n}-i}^{\left(n\right)}=c_{2^{n}+i}^{\left(n\right)},\;i=1,2,\ldots 2^{n}-1\;$$

and

(43) $$\begin{array}{cc} \; & c_{i}^{\left(n\right)}=c_{i}^{\left(n-1\right)},\;i=1,2,\ldots ,2^{n-1}\\ \; & c_{2^{n-1}+i}^{\left(n\right)}=c_{2^{n-1}+i}^{\left(n-1\right)}+c_{i}^{\left(n-1\right)},\;i=1,2,\ldots ,2^{n-1}-1 \end{array}$$

Due to the scale factor b=2, we have the following for the number of sites involved in the renormalization:

(44) $$L\left(n\right)=2^{n+1}-1\;,$$

and the total number of terms involving the multiplicities is

(45) $$\sum_{i=1}^{L(n)}c_{i}=3^{n}\;.$$

The average value of the multiplicities is $\bar{c}=3^{n}/L\left(n\right)$, and its typical value has the same type of scaling with *n*:

(46) $$c_{typ}∼{\left(3/2\right)}^{n}\;.$$

Let us denote by $N_{n}\left(c\right)$ the number of terms having the multiplicity *c* after *n* iterations. According to Equation (45), we have

(47) $$\sum_{c=1}^{F(n)}N_{n}\left(c\right)c=3^{n}\;,$$

where the largest multiplicity after *n* iterations is the Fibonacci number: F(n). Equations (46) and (47) follow the scaling behavior of $N_{n}\left(c\right)$ as

(48) $$N_{n}\left(c\right)∼{\left(4/3\right)}^{n}\;.$$

**Figure 2:**
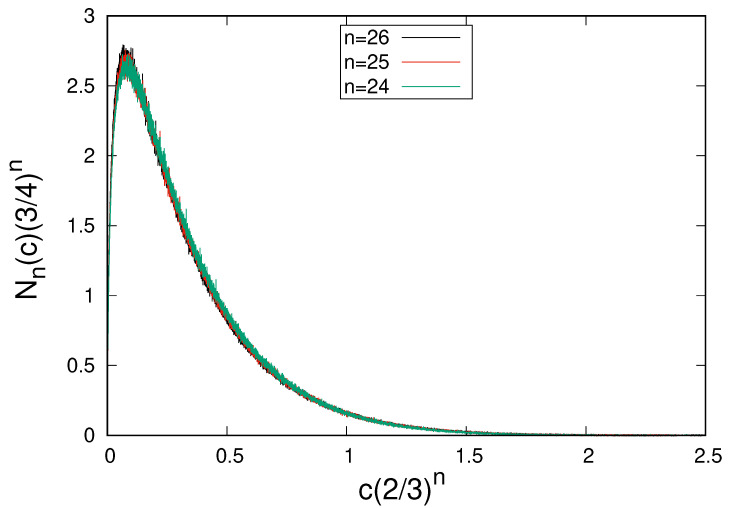
Numerically calculated values of terms with multiplicity *c* versus *c* in scaled variables for different values of the number of iterations *n*. In order to reduce noise, we averaged ten neighboring values.

The numerically calculated values of $N_{n}\left(c\right)$ versus *c* in scaled variables are shown in Figure 2. The points for different values of *n* fit well on a master curve, confirming the scaling relations in Equations (46) and (48).

Let us express the value of the renormalized ratio in Equation (40) as

(49) $$\begin{array}{cc} \; & \ln K^{R(n)}=\sum_{c=1}^{F(n)}c\sum_{k=1}^{N(c)}\ln K_{k}^{\left(c\right)}\approx \\ \; & \sum_{c=1}^{F(n)}c\left[N\left(c\right)\bar{lnK^{\left(c\right)}}+\sqrt{N\left(c\right)\left(Var(\ln J)+Var(\ln h)\right)}v\right]\;. \end{array}$$

Here $\bar{\ln K^{\left(c\right)}}=\bar{\ln J}-\bar{\ln h}=\delta$ is the random quantum control parameter, which is independent of the value of *c* for the dominant part of the sum, and we obtain the combination

(50) $$\ln K^{R(n)}∼3^{n}\delta ∼L^{\ln3/\ln2}\delta \;,$$

thus, the typical correlation-length exponent is

(51) $$\nu_{\mathrm{typ}}=\frac{\ln2}{\ln3}\;.$$

This value is different from that of the random transverse Ising chain with nearest-neighbor interactions, seen in Equation (21).

At the critical point, the fluctuations of the log-couplings grow as $L^{\psi }$, with an exponent:

(52) $$\psi =\frac{3\ln3}{2\ln2}-1=1.377\;,$$

which corresponds to infinite disorder scaling.

We can thus conclude that the critical behavior of the random transverse Ising chain with three-spin couplings is controlled by an infinite disorder fixed point. The obtained value of the typical correlation length exponent indicates that the model belongs to a new infinite disorder universality class.

## 5. Discussion

In this paper, we have considered the transverse Ising chain with two-spin, nearest-neighbor interaction, as well as with three-spin product interaction in the presence of quenched disorder, and studied the quantum critical properties through a block renormalization approach. The two-spin interaction case has been well studied before, and it represents the prototype system having an infinite disorder fixed point with several presumably exact results. In this case, the new feature of our study is that the renormalization is performed analytically for any size of the block, thus the results are correct in the large block limit. Indeed, the obtained results, both in the vicinity of the critical point and in the Griffith’s phase, are in agreement with the previously known exact results.

On the other hand, the model with three-spin couplings represents *terra incognita*; to our knowledge, no random quantum systems with multi-spin interactions had been studied previously. In this case, the renormalization is performed analytically for the block-size b=2. By iterating the transformation, we noticed that the parameters at the sizes of the original model enter several times in the expression of the renormalized one. This is a new feature that is connected to the multispin topology of the interaction. We have calculated the position of the random critical point and demonstrated that the critical properties of the model are controlled by an infinite disorder fixed point. We have also determined the typical correlation-length critical exponent, which turned out to have a different value from that of the two-spin coupling model. The latter, however, represents a very broad universality class, involving models having a discrete order parameter and two-spin interactions.

The renormalization approach we used contains approximations; however, we may expect that some of our results are asymptotically correct for an infinite disorder fixed point. Our investigations can, in principle, be improved by using larger blocks in the transformation; in which case, however, one should involve numerical calculations. With larger blocks, the topology of the iterated renormalization process would remain the same: the original parameters of a given site appear multiple times in the similar way, as shown for b=2 in this paper. Therefore, we believe that the universality class of the random model with three-spin couplings is different from that having two-site interactions.

The studies presented in this paper can be extended in several directions. One could try to apply the traditional SDRG method [4,5] by eliminating successively local degrees of freedom and explore how the topology of the renormalization equations takes a similar form, as obtained in this paper by the block renormalization approach. Another way is to perform numerical investigations of the random three-spin coupling model and check the critical properties. By performing a numerical study, other critical parameters (magnetization exponent, average correlation length exponent, etc.) and Griffith’s singularities can be investigated. Studying multispin models with m>3 could also be interesting, since these probably represent new universality classes. One can also think to generalize the block renormalization approach to higher dimensions, a type of study that has been quite successful for the two-spin interacting model [50,51].

This work is dedicated to the memory of Ralf Kenna. Ralf was an excellent physicist and, among others, he studied the critical behavior of systems above the upper critical dimension [62], which, in our problem, seems to be infinity. How the phase-transition takes place in our models at infinite dimension is still an open question.

## Data Availability

The data presented in this study are available on request from the corresponding author.
